# A Fully Automated Web-Based Program Improves Lifestyle Habits and HbA1c in Patients With Type 2 Diabetes and Abdominal Obesity: Randomized Trial of Patient E-Coaching Nutritional Support (The ANODE Study)

**DOI:** 10.2196/jmir.7947

**Published:** 2017-11-08

**Authors:** Boris Hansel, Philippe Giral, Laetitia Gambotti, Alexandre Lafourcade, Gilbert Peres, Claude Filipecki, Diana Kadouch, Agnes Hartemann, Jean-Michel Oppert, Eric Bruckert, Michel Marre, Arnaud Bruneel, Emilie Duchene, Ronan Roussel

**Affiliations:** ^1^ Department of Endocrinology, Diabetology, Nutrition Hôpitaux Universitaires Paris-Nord Val de Seine Assistance Publique-Hôpitaux de Paris Paris France; ^2^ Paris Diderot Université Sorbonne Paris Cité Paris France; ^3^ Centre de Recherche des Cordeliers Institut National de la Santé et de la Recherche Médicale Paris France; ^4^ Department of Endocrinology Hôpital Pitié-Salpêtrière Paris France; ^5^ Institute of Cardiometabolism and Nutrition Paris France; ^6^ Université Pierre et Marie Curie Université de Paris Sorbonne Universités Paris France; ^7^ L'Unité de Recherche Clinique Hôpital Pitié-Salpêtrière Paris France; ^8^ Department of Nutrition Hôpital Pitié-Salpêtrière Paris France; ^9^ Department of Diabetology Hôpital Pitié-Salpêtrière Paris France; ^10^ Department of Biochemistry Hôpitaux Universitaires Paris-Nord Val de Seine Assistance Publique-Hôpitaux de Paris Paris France; ^11^ Département Hospitalo-Universitaire Fibrosis, Inflammation, Remodelling Paris France

**Keywords:** e-health, nutrition, type 2 diabetes

## Abstract

**Background:**

The prevalence of abdominal obesity and type 2 diabetes mellitus (T2DM) is a public health challenge. New solutions need to be developed to help patients implement lifestyle changes.

**Objective:**

The objective of the study was to evaluate a fully automated Web-based intervention designed to help users improve their dietary habits and increase their physical activity.

**Methods:**

The Accompagnement Nutritionnel de l’Obésité et du Diabète par E-coaching (ANODE) study was a 16-week, 1:1 parallel-arm, open-label randomized clinical trial. Patients with T2DM and abdominal obesity (n=120, aged 18-75 years) were recruited. Patients in the intervention arm (n=60) had access to a fully automated program (ANODE) to improve their lifestyle. Patients were asked to log on at least once per week. Human contact was limited to hotline support in cases of technical issues. The dietetic tool provided personalized menus and a shopping list for the day or the week. Stepwise physical activity was prescribed. The control arm (n=60) received general nutritional advice. The primary outcome was the change of the dietary score (International Diet Quality Index; DQI-I) between baseline and the end of the study. Secondary endpoints included changes in body weight, waist circumference, hemoglobin A1c (HbA1c) and measured maximum oxygen consumption (VO2 max).

**Results:**

The mean age of the participants was 57 years (standard deviation [SD] 9), mean body mass index was 33 kg/m² (SD 4), mean HbA1c was 7.2% (SD 1.1), and 66.7% (80/120) of participants were women. Using an intention-to-treat analysis, the DQI-I score (54.0, SD 5.7 in the ANODE arm; 52.8, SD 6.2 in the control arm; *P*=.28) increased significantly in the ANODE arm compared to the control arm (+4.55, SD 5.91 vs -1.68, SD 5.18; between arms *P*<.001). Body weight, waist circumference, and HbA1c changes improved significantly in the intervention.

**Conclusions:**

Among patients with T2DM and abdominal obesity, the use of a fully automated Web-based program resulted in a significant improvement in dietary habits and favorable clinical and laboratory changes. The sustainability of these effects remains to be determined.

**Trial Registration:**

ClinicalTrials.gov NCT02343107; http://clinicaltrials.gov/ct2/show/NCT02343107 (Archived by WebCite at http://www.webcitation.org/6uVMKPRzs)

## Introduction

Obesity and type 2 diabetes mellitus (T2DM) are public health issues that are growing worldwide, and their incidence rates are closely correlated [1]. Abdominal obesity is a risk factor for T2DM, but is also an independent risk factor for cardiovascular disease; the hypothetical underlying mechanisms are an abnormal adipokine profile and increased insulin resistance [2]. Lifestyle changes that are designed to achieve a healthy diet, weight reduction, and increased physical activity are the cornerstone of the treatment of obese patients with T2DM [3]. Experts recommend individually tailored care for these patients [3]. However, a high-intensity, multidisciplinary intervention (as recommended) is often impossible to implement in real life environments due to limited human resources and the high costs of long-term care. In addition, geographically isolated patients cannot easily access face-to-face education programs. Therefore, it is necessary to develop innovative approaches to improve the adoption of a healthy lifestyle.

Remote counseling using innovative technologies is a promising tool to provide advice and monitor progress at a lower cost than face-to-face education when extended follow-up is needed. A recent review identified 13 randomized trials assessing the value of remote e-coaching for patients with T2DM [4]. The results were generally positive with programs that included an intervention on physical activity, nutrition (individualized goals based on dietary recommendations for people with T2DM and obesity), self-monitoring, or weight loss. However, most of the programs assessed included important human support. In addition, the studies did not comprehensively measure the effects on dietary habits, physical activity, and metabolic parameters. Fully automated Web-based interventions could more easily be proposed on a large scale compared to telehealth programs that require expensive human support. However, few randomized studies (consisting mainly of sending short messages) have comprehensively examined the effects of such programs for T2DM [5,6].

The Accompagnement Nutritionnel de l’Obésité et du Diabète par E-coaching (ANODE) tool is a fully automated Web-based program designed to help users improve their dietary habits and increase their physical activity, with an expected moderate 3-5% weight loss. ANODE includes an interactive two-way program that supports compliance with guidelines for management of obesity and T2DM. In this study, we assessed the efficacy of the ANODE program in patients with T2DM and abdominal obesity by assessing not only changes in dietary habits and physical activity, but also changes in cardiometabolic risk factors. Consequently, the primary endpoint was a quality dietary score, and secondary endpoints were parameters related to metabolism and aerobic fitness.

## Methods

### Study Design and Patients

**Figure 1:**
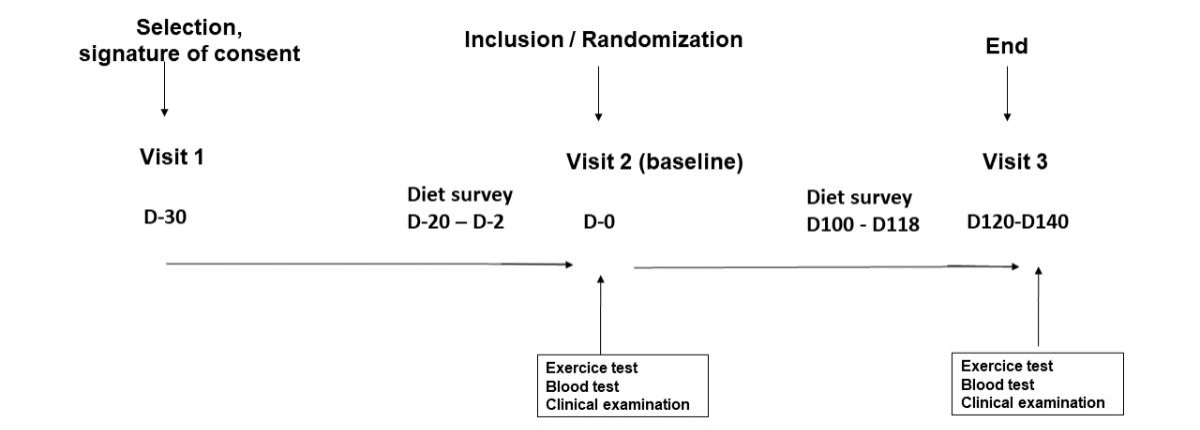
Study design.

The ANODE study was a randomized, comparative effectiveness clinical trial conducted between March 2014 and December 2015 in two university hospitals in Paris, France. Participants were recruited via media advertising or referred directly by their caregivers. Male or female subjects, aged 18-75 years, with abdominal obesity (defined as waist circumference >102 cm for men and >88 cm for women) and T2DM with hemoglobin A1c (HbA1c) >5.6% and <8.5% at the screening visit were included. In addition, patients were eligible if they had already received standard nutritional education for the treatment of diabetes, and if they had been on a stable, nonrestrictive diet for the past 3 months (weight change <4 kg peak-to-trough). Antidiabetic, antihypertensive, and lipid-lowering therapies had to be stable for at least 3 months. Participants had to be covered by French national health insurance. Internet access with frequent use (at least three times per week) was required, as well as an email address and fluent understanding of written and spoken French language. Patients with symptomatic cardiovascular disease, patients requiring rapid control of diabetes, those receiving general or local treatment likely to interfere with assessment of the primary endpoint, and subjects with any severe or acute illness likely to influence the results of the study (or possibly be life-threatening in the short term) were excluded. Patients who had undergone obesity surgery and those with an initial calorie intake <1200 kcal/24 hours or >4000 kcal/24 hour were also excluded. Participants in both arms were invited to attend a screening visit (first visit), an inclusion plus randomization visit (second visit), and a third and final visit at 4 months (Figure 1).

The second and third visits included a clinical examination, an exercise test, and fasting blood collection. Patients provided their written informed consent. The study was approved by the Ethics Committee, Comité de Protection des Personnes Ile-de-France VI, in Paris, France.

#### Randomization

Randomization was performed by means of a computerized randomization program accessed via a secure Web interface. Patients were randomly assigned in a 1:1 ratio to the intervention or control arms. The randomization list was computer-generated and balanced by blocks of undisclosed size. Patients were informed of their allocation by telephone and patients allocated to the intervention arm were given a personal code to connect to the e-coaching program. Control subjects were asked to continue their usual follow-up with their general practitioner and/or specialist.

#### Intervention

The ANODE e-coaching program is a Web-based nutritional support tool developed and provided by the MXS Company. ANODE is designed to improve lifestyle habits, including both diet and physical activity, and consists of four modules: (1) diet and physical activity self-monitoring module, (2) nutritional assessment, (3) balanced diet menu generator, and (4) physical activity education and prescription program. Participants had to complete the questionnaires in the diet and physical activity self-monitoring module for one week to access the other three modules. Participants then accessed the four modules simultaneously, and were asked to connect at least once per week. Human contact was limited to hotline support in cases of technical issues. Patients in the intervention group had unlimited access to the ANODE program, free of charge. No monetary incentives were offered. Outlines of the ANODE e-coaching program are presented in Multimedia Appendix 1 **.** At the time of the study, the program was designed to run only on personal computers.

##### Diet and Physical Activity Self-Monitoring Module

The aim of this module was first to collect information on participants’ habits but also to keep them aware of their dietary intake and of their physical activity level. The computerized dietary survey has been used in epidemiological studies such as the French National Nutrition and Health Study [7], and was designed for self-administration based on a secured user-friendly interface, as described by Lassale et al [8]. Participants reported all foods and beverages (type and quantity) consumed during 24 hours from midnight to midnight. Participants first entered a list of every food item consumed that they could recall via one of the following two options: a food browser (foods grouped by category) or a search engine that accepts spelling errors. Participants then estimated portion sizes of foods with the help of pictures, derived from a previously validated booklet which represents more than 250 generic foods (corresponding to more than 2000 specific food items), each proposed in three different portion sizes. With two intermediate and two extreme quantities, there are seven choices of amounts. Participants could also directly enter the quantity of foods consumed in grams or a measure of volume; they could also use units of purchase (eg, one piece, one bottle, one bar) or standard household units (eg, teaspoons and tablespoons). Physical activity was collected as the daily number of steps measured with a pedometer, and as the self-reported duration and frequency of endurance activities. Participants were encouraged to enter information for each day of the week in order to further personalize the program (see prescription program below).

##### Nutritional Assessment

Based on 24-hour dietary recall, the program informed the patients about the mean level of calories ingested as well as the mean fat, saturated fat, protein, salt, and carbohydrate contents of their diet. Intakes of certain food groups (ie, fish, starchy foods, high-fat foods, dairy products, alcoholic beverages, and water) were also reported [8]. The program also provided advice to ensure a balanced diet according to national guidelines. The more the subject completed the dietary survey (diet and physical activity self-monitoring module), the more the nutritional assessment was precise and advices were personalized.

##### Balanced Diet Menu Generator

This innovative program can construct daily or weekly menus complying with the recommendations of the National Nutrition and Health Program. These menus were: (1) customized to meet the user’s preferences, tastes in food, caloric level, and needs; and (2) practical, as the tool proposed daily or weekly menus adapted to the season and the selected price range (3 levels). Daily or weekly shopping lists and recipes were proposed. Participants were encouraged to use the generator at least three times per week. However, even if he/she did not precisely follow the proposed meals, he/she was advised to draw inspiration from the proposed menus.

##### Physical Activity Education and Prescription Program

The objectives of this program were defined according to a stepwise approach. Five different videos explained how to perform the recommended physical activity: (1) how to perform exercise at the optimal intensity, (2) how to use a pedometer, (3) description of the 5 categories of exercise recommended in the program, (4) description of the intensity of physical activity, and (5) the step test. Each video lasted approximately 2 minutes and was accompanied by a technical sheet. If data were completely collected (see *diet and physical activity self-monitoring module* above) the program was able to fix adjusted quantitative objectives for the number of steps and for the volume (number and duration of the sessions of exercise) of endurance activities. The types of exercise (eg, cycling, brisk walking, running) and the days of the sessions were determined by the participant.

#### Control Arm

Participants assigned to the control arm were asked to continue their usual follow-up with their general practitioner and/or specialist.

### Trial Procedures and Outcomes

After the selection visit, all participants were assessed at baseline (randomization visit) and 4 months later at a final visit.

### Diet and Physical Activity Assessment

Diet was evaluated by a 3-day dietary recall (two weekdays and one weekend day). This assessment was performed before the randomization visit and during the two weeks preceding the final visit. The dates of the 3-day dietary recall were determined randomly in advance by the study team dietician. When one or more of the 3-day dietary recalls could not be completed, another three dates were randomly determined. Dietary recalls were performed with an interactive Web-based self-administered dietary diary [9] but data were verified and completed by phone with a dietician blinded to the patient’s allocation. The primary endpoint of the study was the change in the dietary score (International Diet Quality Index, DQI-I [10,11]) between baseline and the end of the study. The DQI-I score (range 0-100) comprises four components (variety, adequacy, moderation, and overall balance) and the cutoffs used for adequacy and moderation were those corresponding to French recommendations [12]. Table 1 summarizes the characteristics of the DQI-I score; detailed information on computation of the DQI-I dietary score can be found elsewhere [11]. The amount of physical activity was evaluated using the short version of the International Physical Activity Questionnaire (IPAQ) [13] completed online by the subjects before randomization and at the final visit. Due to a technical issue, sitting time (although part of the IPAQ questionnaire) was not recorded during the trial.

### Clinical Measurements

Body weight was measured to the nearest 0.1 kg, with participants fasting and clothed without shoes, using automated digital scales (TANITA T6360, Tanita Co., Tokyo, Japan). Body mass index (BMI) was calculated as the ratio between weight and height squared with weight expressed in kilograms and height expressed in meters. Waist circumference was measured with an inelastic tape to the nearest 0.1 cm at the level of the iliac crest during shallow breathing.

### Laboratory Assessments

Total cholesterol, high-density lipoprotein-cholesterol (HDL-C) and triglyceride concentrations were determined by automated enzymatic methods. Low-density lipoprotein (LDL-C) was calculated using Friedewald’s equation for triglycerides <340 mg/dl (3.9 mmol/L), or measured directly for triglycerides >340 mg/dl (3.9 mmol/L) but <700 mg/dl (8 mmol/L). Blood samples were drawn for assays of creatinine, uric acid, liver enzymes, fasting glucose, and HbA1c as part of routine care. Systemic inflammation was assessed by the plasma concentration of high-sensitivity C-reactive protein (hs-CRP).

### Measurement of Aerobic Fitness

Aerobic fitness was determined by maximum oxygen consumption (VO_2_ max), measured by an incremental cardiopulmonary exercise test on a cycle ergometer, with increments of 15-30 Watts/2 minutes [14]. Oxygen consumption was expressed as VO_2_ per kg total body weight per minute (mL/kg/minute). We tried to achieve maximal cardiopulmonary exercise testing by using strong verbal encouragements. After exhaustion, active then passive recoveries were recorded with the patient seated.

**Table 1:** Characteristics and computation of the DQI-I diet quality scores [10,11].

Components	Score range: 0-100 points
Variety	Overall food group variety (0-15 points); within-group variety for protein source (0-5 points)
Adequacy	Vegetables, fruits, cereals, fiber, protein, iron, calcium, vitamin C (0-5 points each); nutritional recommendations are country-specific (in this case France)
Moderation	Total fat, saturated fat, cholesterol, sodium, empty-energy foods (0-6 points each)
Overall balance	Macronutrient ratio (carbohydrate : protein : fat, 0-6 points); fatty acid ratio (polyunsaturated fatty acid : monounsaturated fatty acid : saturated fatty acid, 0-4 points)

### Satisfaction Questionnaire

In a post hoc analysis, we collected some information about the subjective opinion of the patients in the intervention group. The questionnaire was sent by email. Patients were asked if they would recommend the program and if they encountered difficulties with the use of the platform. In addition, respondents were encouraged to send comments about the quality of the program.

### Required Number of Subjects

To show a 3-point difference in the variation of the DQI-I scores between the two arms over a 4-month period (with an SD of 5, a statistical power of 80%, and a type 1 error of 5% using a two-tailed test), the required number of subjects was estimated to be 45 per arm. Sixty subjects were included in each arm to anticipate patients lost to follow-up.

### Statistical Analysis

Baseline characteristics in each study arm were expressed as frequencies and percentages for categorical variables, and as means and standard deviations for continuous variables. Changes in DQI-I scores (primary outcome) were compared between the two randomization arms using student t-tests. In cases of missing data, changes in DQI-I score were imputed to 0 (no variation). Other continuous variables were compared using student t-tests or Wilcoxon tests, and categorical variables were compared using the Chi-square or Fisher’s exact tests, as appropriate. To assess whether the effect of the ANODE program on the DQI-I score varied according to baseline characteristics, post hoc subgroup analyses were performed according to age, sex, HbA1c, BMI, education level, and occupation. In these analyses, continuous variables were dichotomized according to the median, and interaction tests were performed. Finally, in addition to the intention-to-treat analysis, a per-protocol analysis was also performed, using only complete data. All analyses were conducted with the two-sided alpha risk of 5%, and were performed using SAS software version 9.2.

## Results

### Participants

**Figure 2:**
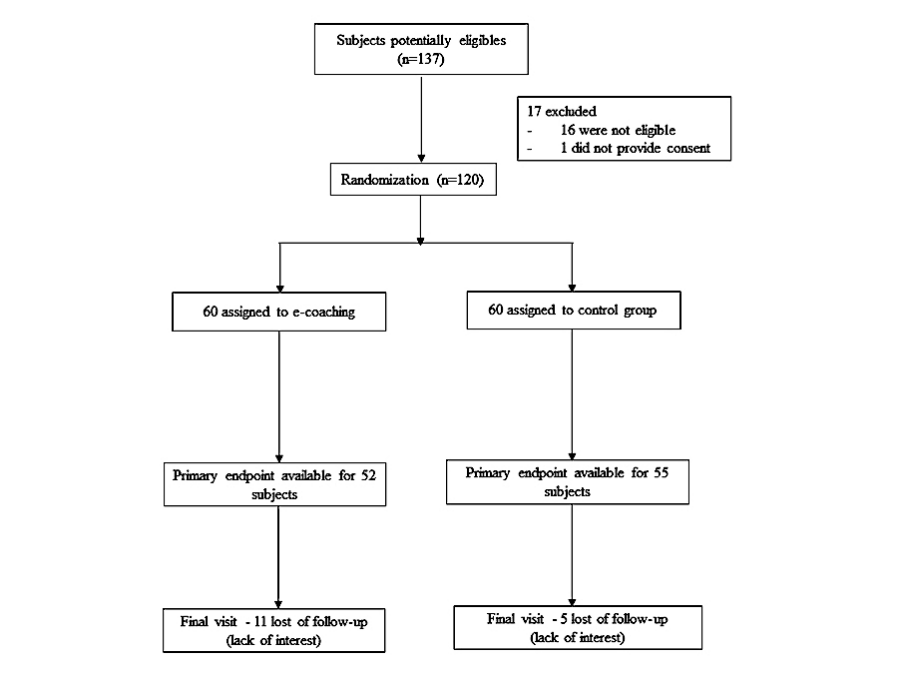
Flowchart of the study.

Figure 2 shows the flow chart of participants. Among the volunteers who contacted the study team for participating in the study, 363 were subsequently contacted by phone and 137 were considered eligible. After the exclusion of 17 subjects who eventually did not meet all criteria (n=16) or who finally decided not to participate (n=1), 120 subjects were enrolled and randomized. At the end of the study, 13 subjects (8 in the intervention arm and 5 in the control arm) were unable to be contacted to collect dietary data, and 17 subjects (12 in the intervention arm and 5 in the control arm) did not attend the last visit. The primary endpoint was available for 107 of 120 (89.2%) randomized subjects.

Baseline characteristics were similar between the two arms (Multimedia Appendix 2). The majority of participants were females (80/120, 66.7%). On average, participants were moderately obese with no difference in baseline DQI-I scores. Intake of the main nutrients was comparable between arms.

### Dietary Changes

Dietary characteristics of the participants are presented in Multimedia Appendix 2. The DQI-I score was 54.0 (SD 5.7) in the e-coaching arm and 52.8 (SD 6.2) in the control arm (*P*=.28 between arms). The intention-to-treat analysis at 16 weeks showed that e-coaching resulted in a significant improvement in the DQI-I score: +4.55 (SD 5.91) in the intervention arm and -1.68 (SD 5.18) in the control arm (*P*<.01 between arms; Table 2).

Moreover, changes in dietary intake tended to differ between arms for lipids (*P*=.02), saturated fats (*P*<.01), sodium (*P*=.07), and empty calories (*P*=.06), always towards healthier foods in the intervention arm (Table 3).

### Changes in Anthropometric Variables, Cardiometabolic Risk Factors, and Aerobic Fitness

**Figure 3:**
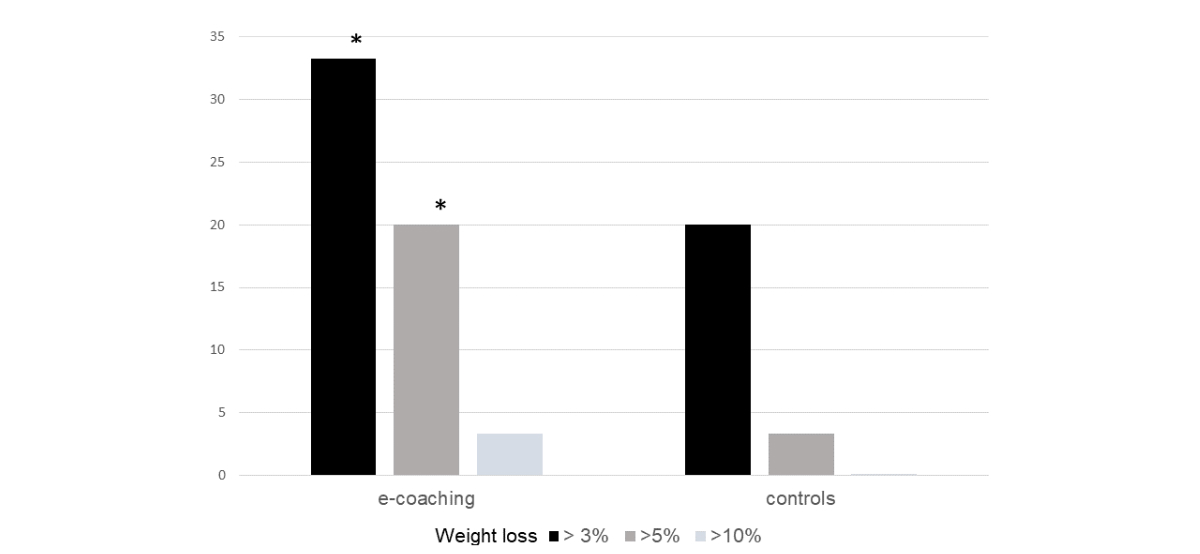
Percentage of patients in the intervention and control arms achieving weight loss >3%, >5% or >10% at 16-week follow-up, in the intention-to-treat population. *P<.05 between intervention and control arms.

Compared to usual care, the ANODE program was associated with reduced body weight, waist circumference, and HbA1c (Multimedia Appendix 2). A significantly higher proportion of e-coaching subjects achieved weight loss >3% (20/60, 33.3% of e-coaching subjects vs 4/60, 6.7% of control subjects on intention-to-treat analysis; *P*<.01) and weight loss >5% (12/60, 20.0% vs 2/60, 3.3% on intention-to-treat analysis; *P*<.01; Figure 3). Only two subjects (2/60, 3.3%) in the ANODE arm and no subject in the control arm achieved >10% weight loss, respectively (*P*=.15 between arms).

No significant differences in terms of change in blood pressure, plasma lipids, aminotransferases, gamma glutamyl aminotransferase, uric acid, fasting glucose, VO_2_ max or hs-CRP were observed between the two arms at 4 months (Table 4). No significant changes in physical activity assessed by the IPAQ questionnaire were observed in the intervention arm compared to control subjects (data not shown).

**Table 2:** Main primary and secondary endpoints in the intention-to-treat analysis.

Parameter	Mean difference (95% CI)			*P*-value between arms
	e-coaching (n=60)	Control (n=60)		
DQI-I score	4.55 (5.91)	-1.68 (5.18)	<.001	
Energy, kcal/day	-271 (648)	-26 (663)	.11	
Fibers, g/day	3.3 (12.9)	-0.7 (9.1)	.16	
Carbohydrates, g/day	-24 (74)	4 (95)	.08	
Proteins, g/day	-5.8 (29)	-6.8 (33)	.87	
Lipids, g/day	-15.4 (34.1)	1.0 (39.3)	.02	
Saturated fat, g/day	-8.6 (15.0)	3.1 (18.2)	<.001	
Sodium, mg/day	-442 (1366)	175 (1981)	.07	
Calcium, mg/day	-20.3 (347.3)	52.2 (428.4)	.31	
Empty calories, kcal/day	-307 (663.2)	-84.6 (639.2)	.06	
Body weight, kg	-2.3 (3.0)	0.2 (2.5)	.01	
Waist circumference, cm	-0.9 (4.7)	0.8 (3.6)	.01	
HbA1c, %	-0.30 (0.94)	0.21 (0.70)	<.001	
VO_2_ max, mL/minute/kg	2.99 (6.20)	2.17 (4.98)	.20	

**Table 3:** DQI-I scores and component changes according to study arms in the intention-to-treat analysis.

Parameter		Score ranges (points)	DQI-I score between arms, mean difference (95% CI)		*P*-value between arms
			e-coaching	Control	
DQI-I score		0-100	4.55 (5.91)	-1.68 (5.18)	<.001
Variety score		0-20	0.28 (1.93)	-0.18 (1.56)	.15
Adequacy score		0-40	1.41 (4.07)	-1.00 (3.90)	.001
Moderation score		0-30	2.55 (3.43)	-0.38 (3.11)	<.001
Overall balance score		0-10	0.31 (1.04)	-0.11 (0.60)	.01
**DQI-Components**					
	Vegetable	0-5	0.33 (1.04)	-0.28 (1.22)	.01
	Fruit	0-5	0.41 (1.10)	-0.07 (1.18)	.02
	Grain	0-5	0.13 (1.26)	-0.27 (1.28)	.09
	Fiber	0-5	0.60 (0.96)	-0.18 (0.96)	<.001
	Protein	0-5	0.03 (0.19)	0.01 (0.15)	.48
	Iron	0-5	-0.14 (0.98)	-0.20 (0.90)	.75
	Calcium	0-5	0.09 (0.91)	-0.06 (1.02)	.41
	Vitamin C	0-5	-0.04 (1.27)	0.03 (1.47)	.76
	Total fat	0-6	0.50 (0.98)	-0.15 (0.90)	<.001
	Saturated fat	0-6	0.55 (1.11)	-0.16 (0.94)	.02
	Cholesterol	0-6	0.62 (1.57)	-0.20 (2.12)	.02
	Sodium	0-6	0.88 (1.63)	0.20 (1.70)	.03
	Empty-calorie food	0-6	0.00 (0.00)	0.00 (0.00)	1

**Table 4:** Other secondary endpoints in the intention-to-treat analysis.

Parameter	Mean difference (95% CI)		*P*-value between arms	
	e-coaching (n=60)	Control (n=60)		
Fasting blood glucose, mmol/L	-0.14 (1.46)	0.11 (1.57)		.36
Uric acid, µmol/L	-6.37 (45.45)	-8.58 (42.29)		.78
Total cholesterol, mg/dL	0.0 (0.21)	-0.04 (0.32)		.40
LDL-C, mg/dL	0.03(0.20)	-0.02 (0.28)		.43
HDL-C, mg/dL	0.00 (0.05)	0.00 (0.06)		.78
Triglycerides, mg/dL	-0.22 (1.05)	-0.14 (0.50)		.67
hs-CRP, mg/L	-0.24 (1.48)	0.08 (2.01)		.81
Serum glutamic oxaloacetic transaminase, IU/L	-3.52 (10.41)	-0.17 (12.63)		.13
Serum glutamic pyruvic transaminase, IU/L	-1.78 (6.76)	-1.28 (7.61)		.70
Gammaglutamyl-transferases, IU/L	-2.13 (20.62)	2.97 (17.73)		.47

**Table 5:** Main primary and secondary endpoints in the per-protocol analysis

Parameter	Mean difference (95% CI)			*P*-value between arms
	e-coaching	Control		
DQI-I score^a^	5.25 (6.05)	-1.83 (5.38)	<.001	
Energy, kcal/day^a^	-319 (693)	-29 (700)	.09	
Fibers, g/day^a^	4 (14)	-1 (10)	.12	
Carbohydrates, g/day^a^	-28 (80)	4 (101)	.08	
Proteins, g/day^a^	-7 (32)	-8 (35)	.92	
Lipids, g/day^a^	-18 (36)	1 (41)	.01	
Saturated fat, g/day^a^	-10 (16)	3 (19)	<.001	
Sodium, mg/day^a^	-521 (1470)	195 (2089)	.05	
Calcium, mg/day^a^	-24 (377)	58 (452)	.32	
Empty calories, kcal/day^a^	-361 (706)	-94 (674)	.05	
Body weight, kg^b^	-2.9 (3.1)	0.2 (2.6)	<.001	
Waist circumference, cm^c^	-1.3 (5.6)	0.90 (3.9)	.01	
HbA1c, %^d^	-0.37 (1.04)	0.23 (0.73)	<.001	
VO_2_ max, mL/minute/kg	3.73 (6.74)	2.60 (5.36)	.09	

^a^Available for 52 subjects in the intervention arm and 55 subjects in the control arm

^b^Available for 47 subjects in the intervention arm and 55 subjects in the control arm

^c^Available for 41 subjects in the intervention arm and 50 subjects in the control arm

^d^Available for 48 subjects in the intervention arm and 55 subjects in the control arm

### Satisfaction Questionnaire

Of the 60 subjects of the intervention arm, 55 (92%) returned the satisfaction questionnaire by email. The overall perception of the program was good, as suggested by the comments that patients provided. Seventy percent of patients would recommend using the program for patients like them. No patient declared having encountered difficulties with the use of the platform. All the patients were able to use the platform without major difficulties and they did not need any training.

### Additional Analysis

We collected the number and frequency of connections that participants made with the platform. There was a significant variation in the number of participant connections during the study. During the first month of the intervention, 93% (56/60) of participants logged in at least once per week. This percentage decreased progressively throughout the study, to reach one third of the patients in the final month.

We compared baseline characteristics of completers (subjects who completed the study) and noncompleters. The two groups were similar except for the prescription of antihypertensive drugs, which was significantly more frequent among completers (data not shown).

Interaction tests were performed to explore potential subgroups deriving a greater benefit from the intervention. No interaction was observed with respect to the primary outcome for subgroups defined according to the baseline variables (HbA1c, age, sex, BMI, education level, and occupation). In the per-protocol analysis (only including patients with complete data at the end of the study), more marked differences were observed between the ANODE arm and the control arm, and the improvement in VO_2_ max tended towards statistical significance (Table 5,Table 6, and Table 7). Finally, we performed correlation analyses between the DQI-I score changes and the changes of the main variables of interest. The correlations were statistically significant for HbA1c and body weight (Table 8).

**Table 6:** DQI-I Scores and component changes according to study arms in the per-protocol analysis.

Parameter		Score ranges (points)	DQI-I score between arms, mean difference (95% CI)		*P*-value between arms
			e-coaching (n=52)	Control (n=55)	
DQI-I score		0-100	5.25 (6.05)	-1.83 (5.38)	<.001
Variety score		0-20	0.33 (2.07)	-0.20 (1.63)	.14
Adequacy score		0-40	1.62 (4.34)	-1.09 (4.06)	<.001
Moderation score		0-30	2.94 (3.52)	-0.42 (3.25)	<.001
Overall balance score		0-10	0.36 (1.11)	-0.12 (0.63)	.01
**DQI-Components**					
	Vegetable	0-5	0.38 (1.41)	-0.30 (1.27)	.01
	Fruit	0-5	0.47 (1.17)	-0.07 (1.23)	.02
	Grain	0-5	0.15 (1.36)	-0.29 (1.34)	.09
	Fiber	0-5	0.69 (1.00)	-0.19 (1.00)	<.001
	Protein	0-5	0.04 (0.21)	0.01 (0.16)	.46
	Iron	0-5	-0.17 (1.06)	-0.22 (0.94)	.79
	Calcium	0-5	0.10 (0.97)	-0.06 (1.07)	.41
	Vitamin C	0-5	-0.05 (1.36)	0.04 (1.54)	.76
	Total fat	0-6	0.58 (1.17)	-0.25 (0.84)	<.01
	Saturated fat	0-6	0.63 (1.17)	-0.16 (0.94)	<.001
	Cholesterol	0-6	0.71 (1.67)	-0.22 (2.22)	.02
	Sodium	0-6	1.02 (1.71)	0.22 (1.77)	.02
	Empty-calorie food	0-6	0.00 (0.00)	0.00 (0.00)	1

**Table 7:** Other secondary endpoints in the per-protocol analysis.

Parameter	Mean difference (95% CI)			*P*-value between arms
	e-coaching (n=49)	Control (n=55)		
Fasting blood glucose, mmol/L	-0.18 (1.62)	0.12 (1.66)	.36	
Uric acid, µmol/L	-7.80 (50.27)	-10.30 (46.22)	.80	
Total cholesterol, mg/dL	0.00 (0.24)	-0.04 (0.34)	.43	
LDL-C, mg/dL	0.03 (0.22)	-0.02 (0.29)	.51	
HDL-C, mg/dL	0.00 (0.06)	0.00 (0.06)	.76	
Triglycerides, mg/dL	-0.27 (1.16)	-0.15 (0.53)	.97	
hs-CRP, mg/L	-0.31 (1.67)	0.09 (2.12)	.97	
Serum glutamic oxaloacetic transaminase, IU/L	-4.31 (11.39)	-0.19 (13.32)	.12	
Serum glutamic pyruvic transaminase, IU/L	-2.18 (7.43)	-1.43 (8.02)	.62	
Gammaglutamyl-transferase, IU/L	-2.61 (22.84)	3.30 (18.68)	.38	

**Table 8:** Correlation coefficients between the changes in DQI-I score and HbA1c, body weight, and waist circumference in the ANODE study.

	e-coaching	
	r²	*P*-value
DQI-I vs HbA1c	-0.48	<.001
DQI-I score vs body weight	-0.37	<.001
DQI-I score vs waist circumference	-0.12	.21

## Discussion

The randomized controlled ANODE trial in patients with T2DM and abdominal obesity showed that an automated Web-based program improved dietary habits and was associated with better weight loss and better reduction of waist circumference, compared to usual care. Moreover, glycemic control was also improved. This study is one of the first to test a fully automated but interactive Web-based program to improve lifestyle in T2DM patients. Indeed, most previous studies on telehealth for the management of T2DM have focused on telemonitoring of self-monitored blood glucose and/or were based on e-coaching conducted by phone and/or with single-way programs limited to short text messaging [4,15].

### Comprehensive Analysis of Dietary Changes Associated With an Automated Web-Based Program

Several approaches to scoring dietary habits have been developed. In the present study we used the DQI-I based on 3-day dietary recall, because it measures overall diet quality [11]. To limit under- and over-reporting, self-reported data that were recorded with a validated Web-based tool [9] were verified via phone by a dietician blinded to randomization (and who did not provide any advice). The impact of an automated Web-based program on lifestyle habits, including dietetic changes, has rarely been reported in patients with T2DM. Cotterez et al [16] reviewed Internet interventions to support lifestyle modification for diabetes management. Only five such studies were identified in the literature [5,17-21]. Among them, only one concluded that dietary changes were significantly better for individuals randomized to the Web-based intervention compared to control subjects [18]. The study by Turnin et al [22], which was not included in this review, was conducted before the era of the Internet and tested a computer-assisted diet education system in patients with T2DM. This program achieved a significant improvement of dietetic knowledge and improved dietary habits; it was also associated with a decrease in HbA1c levels compared to usual care. [22]

### Effect on Body Weight and Waist Circumference

A significant reduction in body weight and waist circumference was observed in the current trial. A recent review of technology-based interventions for the treatment of overweight and obese patients summarized data extracted from 27 clinical trials, and revealed the superiority of most of the interventions tested compared to control care [23]. However, unlike the ANODE program, most of these studies included time-consuming human intervention, which is an important element determining the feasibility of practical application. Two studies appeared to use fully automated programs. First, Tanaka et al [24] tested an automated tailored behavioral program. This program resulted in a significant weight loss only among obese subjects. In this subgroup, the weight loss at 3 months (-3.0 kg vs -1.4 kg in the control arm) was comparable to our results [24]. Second, in the study by Tate et al [25], participants randomized to the intervention arm who received automatically tailored messages achieved a similar weight loss at 3 months, compared to the participants randomized to the control arm who received human email counseling [25]. Taken together, these reports and the current study support the efficacy of automated interventions, at least in the intermediate term.

One question raised by the ANODE study is whether the modest weight loss obtained with e-coaching is clinically relevant. Previous modeling by the Nice Institute for Health and Care Excellence showed that at least a 1 kg weight loss among overweight or obese adults is likely to be cost-effective, provided the cost of the intervention is less than £100 and the weight difference is maintained for life [26]. The first condition is likely to be met by the program tested here because no human intervention is necessary. Whether weight loss can be maintained in the long term remains to be confirmed.

### Effect on Physical Activity and Aerobic Fitness

In the ANODE study, we measured aerobic fitness by VO_2_ max, which is an objective measure of aerobic activity performed over time [27,28]. Upon per-protocol analysis, the results revealed a trend towards improvement of aerobic fitness in the ANODE arm compared to the control arm. In contrast, physical activity evaluated by self-reported data was not increased in the intervention arm compared to control subjects in any of the analyses (intention-to-treat or per-protocol). Previous studies have reported conflicting data when the impact of an automated program for physical activity was assessed by questionnaires [29-32]. These questionnaires likely present certain limitations regarding capture of physical activity, especially in obese patients [33]. Another way to assess physical activity is to use trackers such as accelerometers or pedometers. Some studies have used these methods and have demonstrated the superiority of automated remote programs compared to usual care to increase physical activity [34,35]. Future studies in obese and/or patients with T2DM should objectively monitor physical activity with trackers and measure aerobic fitness in addition to self-reported data.

### Limitations and Strengths

The limitations of the study include the absence of follow-up beyond 16 weeks, a relatively small sample size, and an unavoidable open-label design. In addition, only two centers were involved in recruitment. Another limitation is the lack of measurement of physical activity by trackers, although VO_2_ max was measured directly. The strengths of this study include a randomized design and comprehensive analysis of dietary habits and cardiometabolic risk factors.

### Feasibility of Implementing the ANODE Program

As suggested by the satisfaction questionnaire, the program was appreciated by the intervention group and was easy to use. However, because inclusion in the study required, “a frequent use (at least three times per week) of Internet as well as an email address” we cannot extrapolate this data to all patients with T2DM. Our opinion is that the feasibility of implementing such a program would be easy in a population that is accustomed to using the Internet.

### Conclusion

The use of the automated Web-based ANODE e-coaching program in patients with T2DM and abdominal obesity was associated with a significant control-subtracted improvement in diet quality and several important cardiometabolic risk factors. The program can be delivered remotely with limited human resources, and therefore has potential for cost-effectiveness, and subsequently broad dissemination if generalizability and longer-term sustainability are demonstrated.

## Appendix

Multimedia Appendix 1 Outlines of the e-coaching ANODE program.

Multimedia Appendix 2 Baseline characteristics of the population.

## Abbreviations

ANODE: Accompagnement Nutritionnel de l’Obésité et du Diabète par E-coaching
BMI: body mass index
DQI-I: International Diet Quality Index
HbA1c: hemoglobin A1c
HDL-C: high-density lipoprotein cholesterol
hs-CRP: high-sensitivity C-reactive protein
IPAQ: International Physical Activity Questionnaire
LDL-C: low-density lipoprotein cholesterol
SD: standard deviation
T2DM: type 2 diabetes mellitus
VO2max: maximum oxygen consumption
