# Supplementary figures and images for: A Fully Automated Web-Based Program Improves Lifestyle Habits and HbA1c in Patients With Type 2 Diabetes and Abdominal Obesity: Randomized Trial of Patient E-Coaching Nutritional Support (The ANODE Study)
Source: J Med Internet Res. 2017 Nov 8;19(11):e360. doi: 10.2196/jmir.7947 (PMC5700402; doi:10.2196/jmir.7947)

## Slide 1
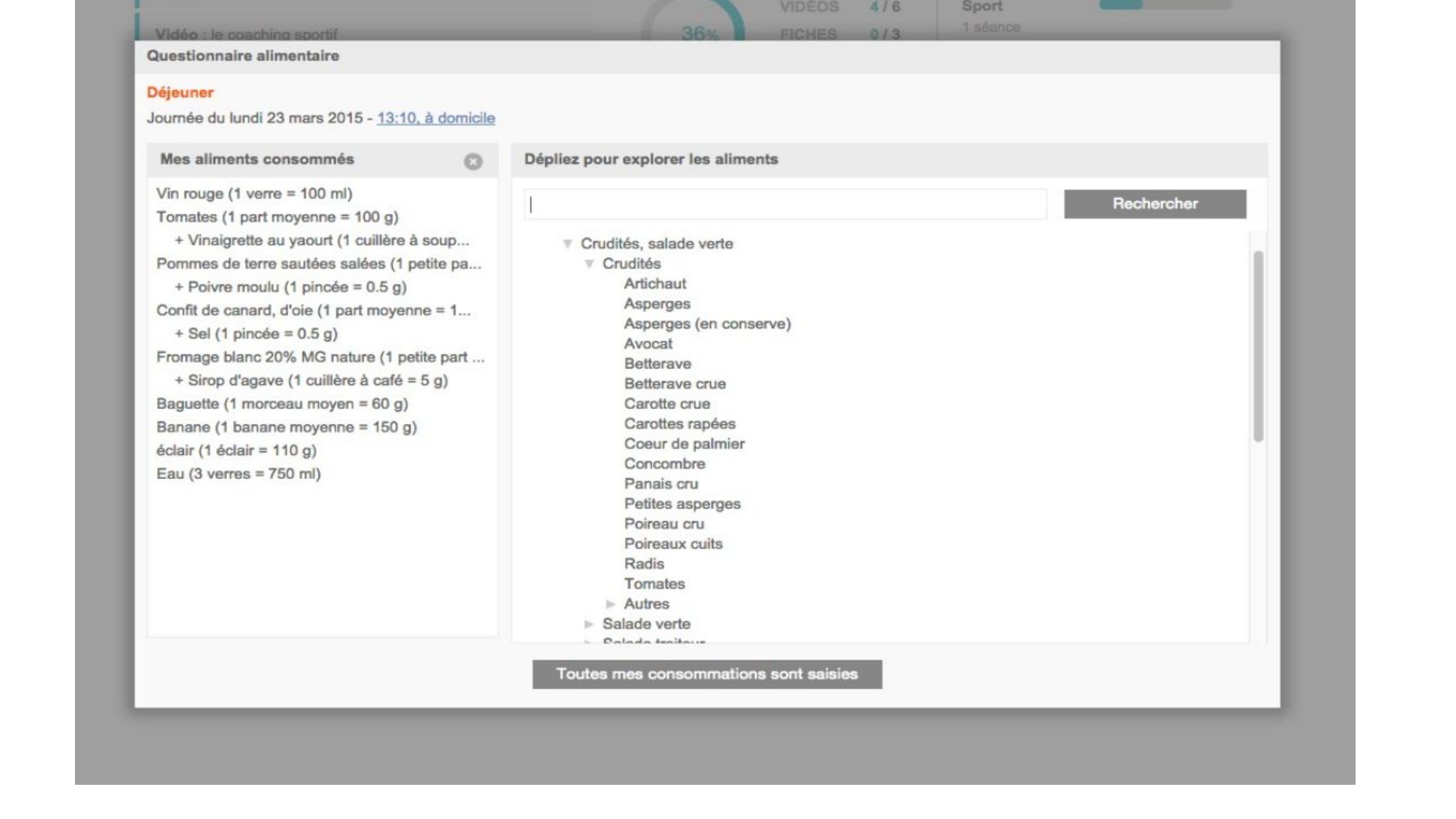

#

## Slide 2
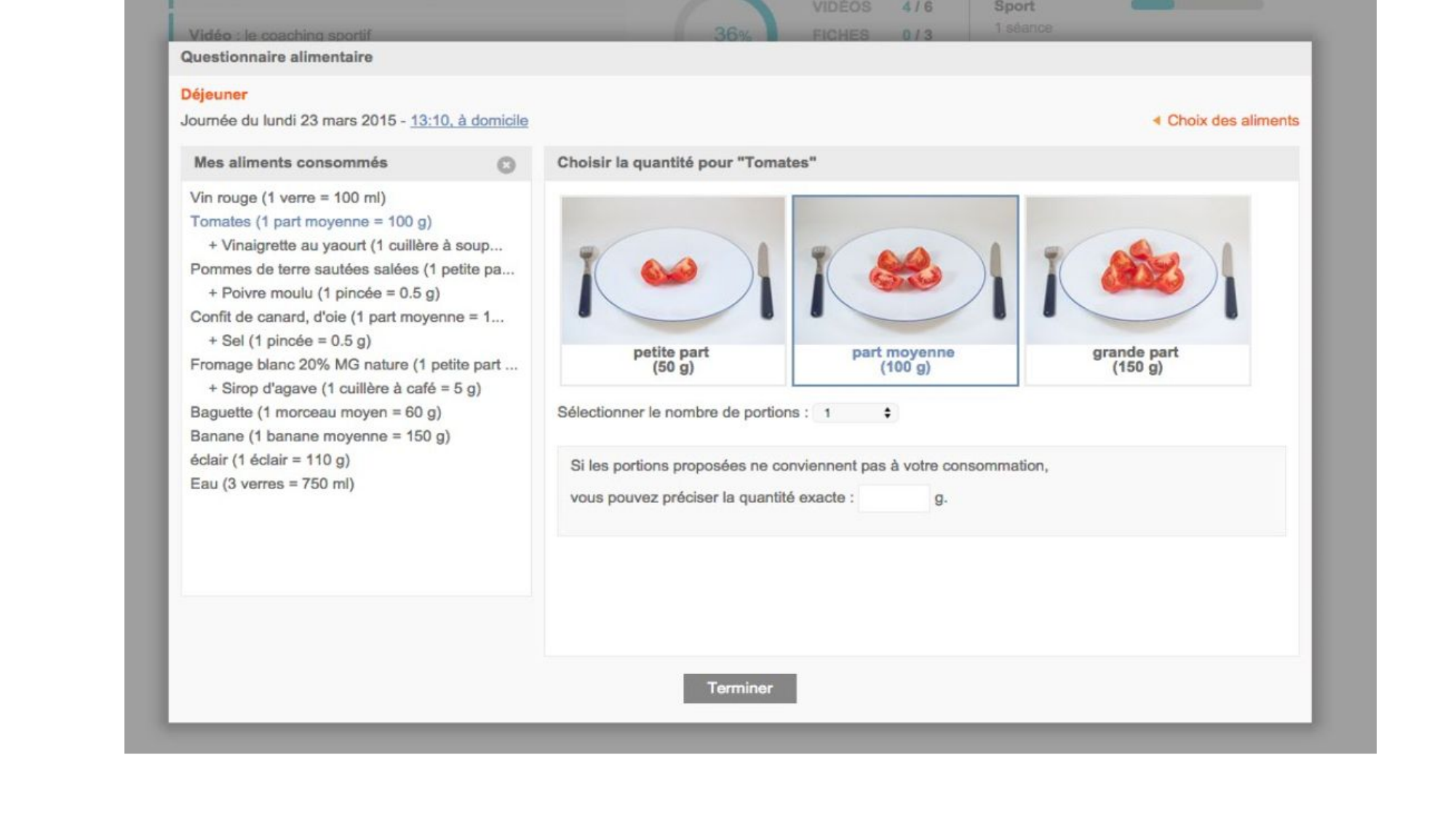

#

## Slide 3
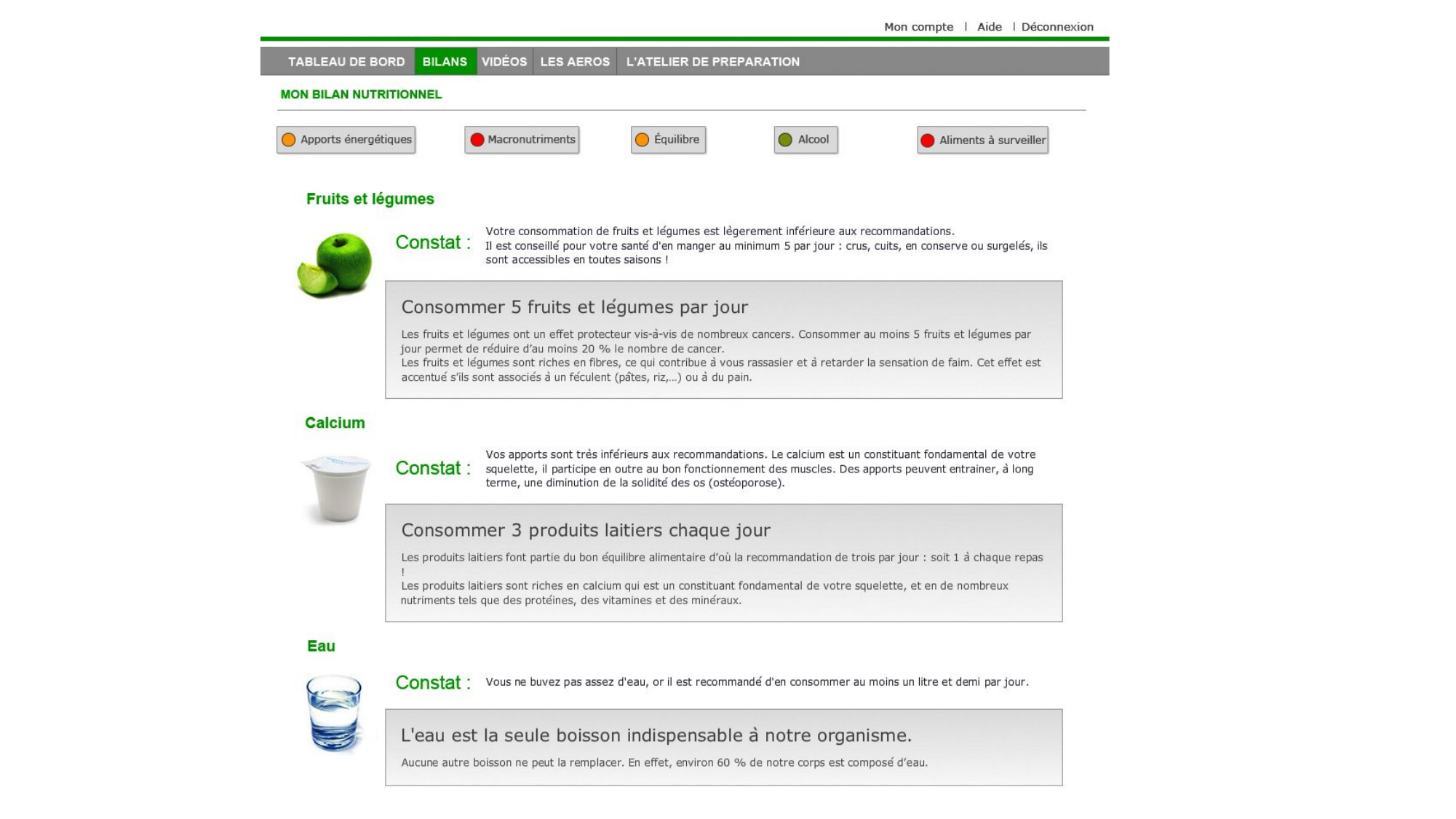

## Slide 4
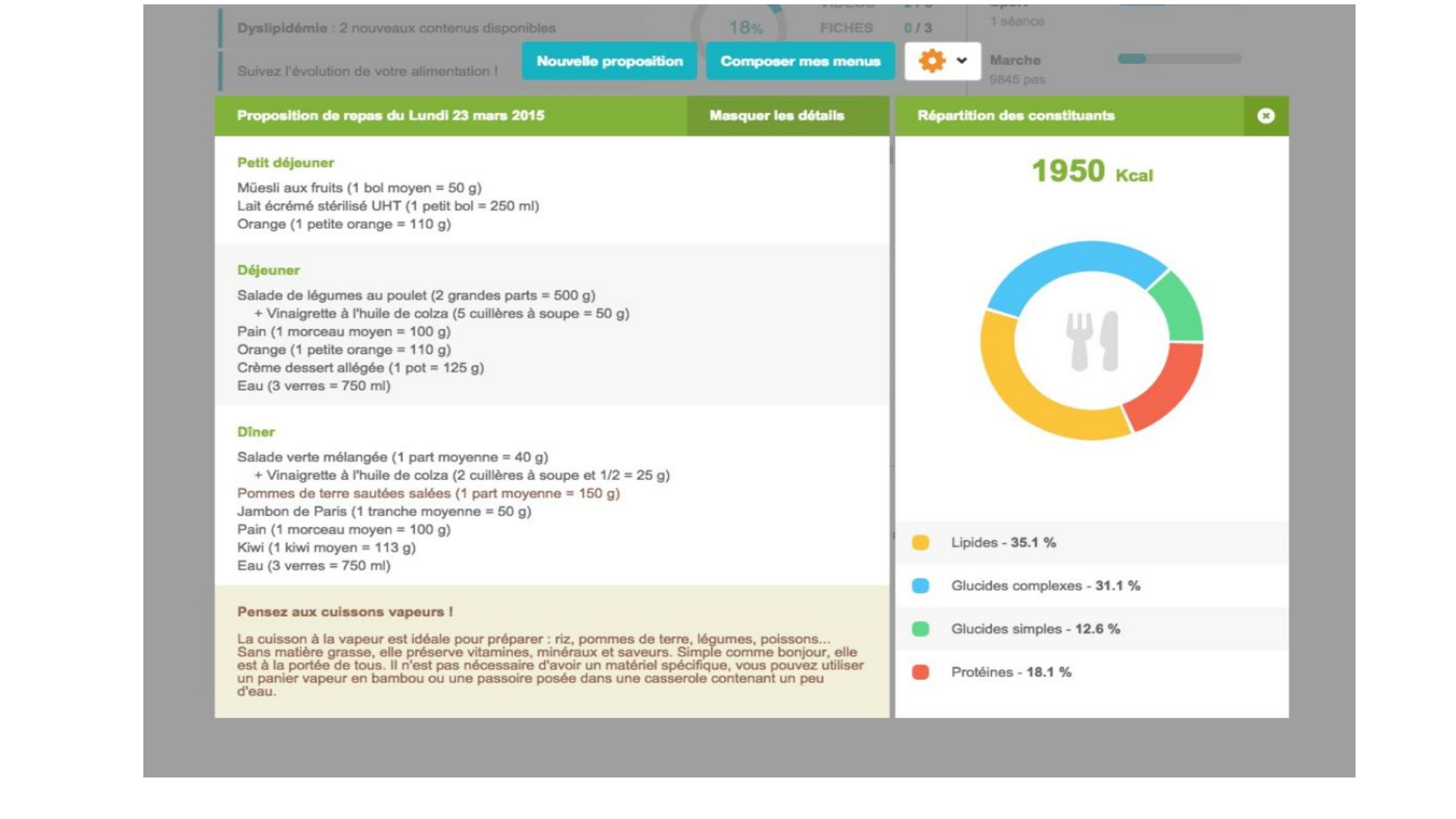

## Slide 5
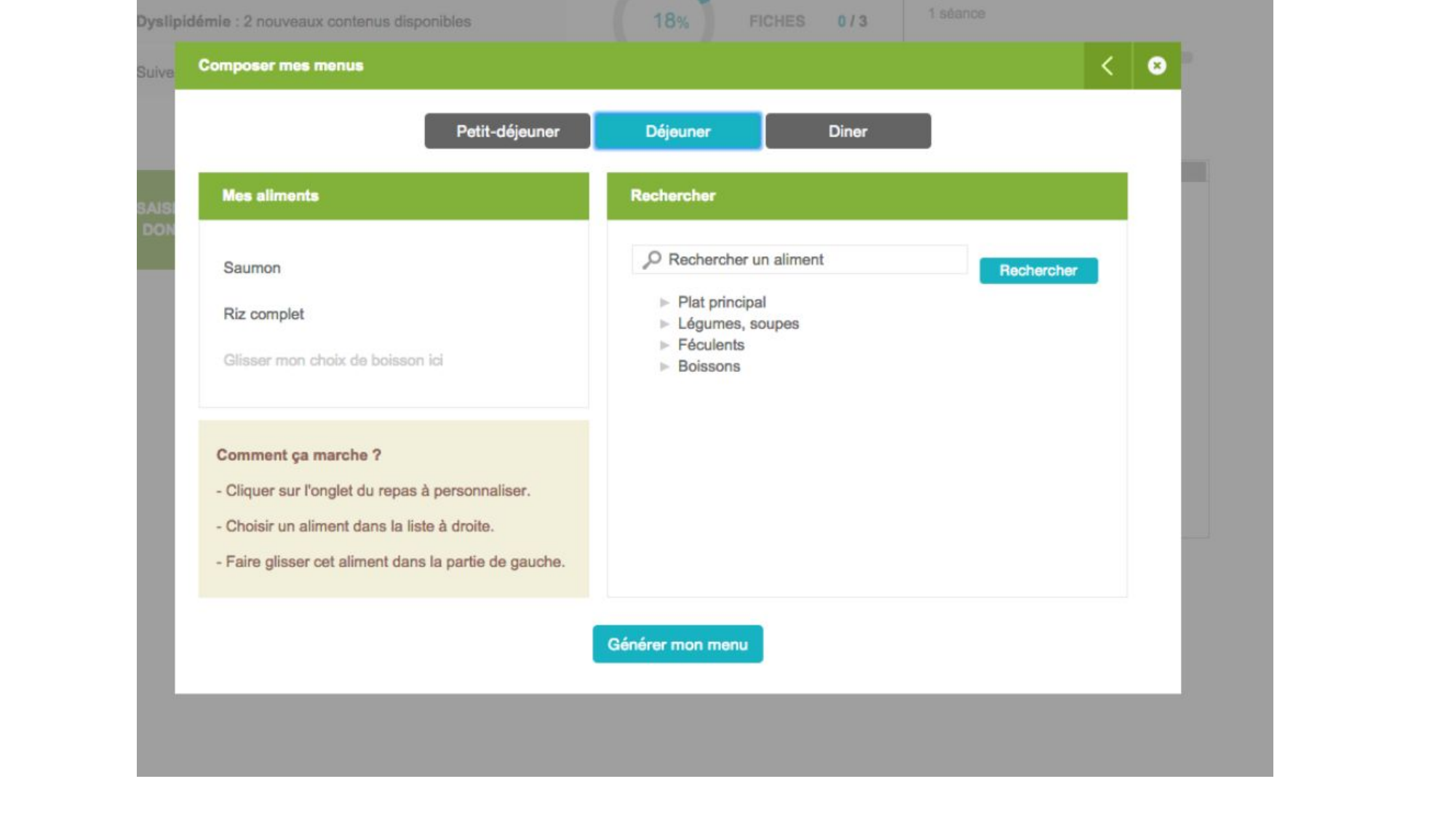

#

## Slide 6
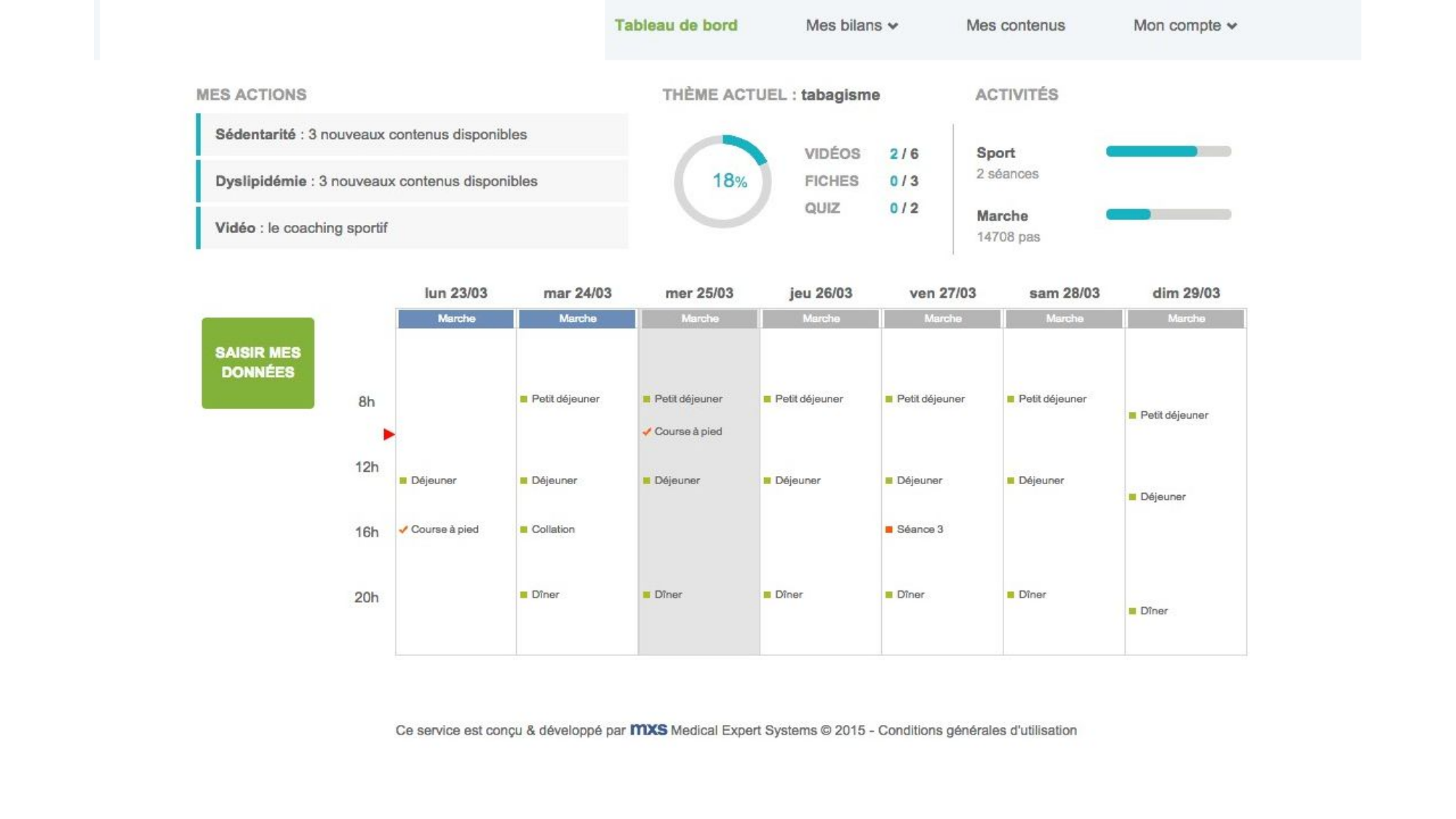

#

## Slide 7
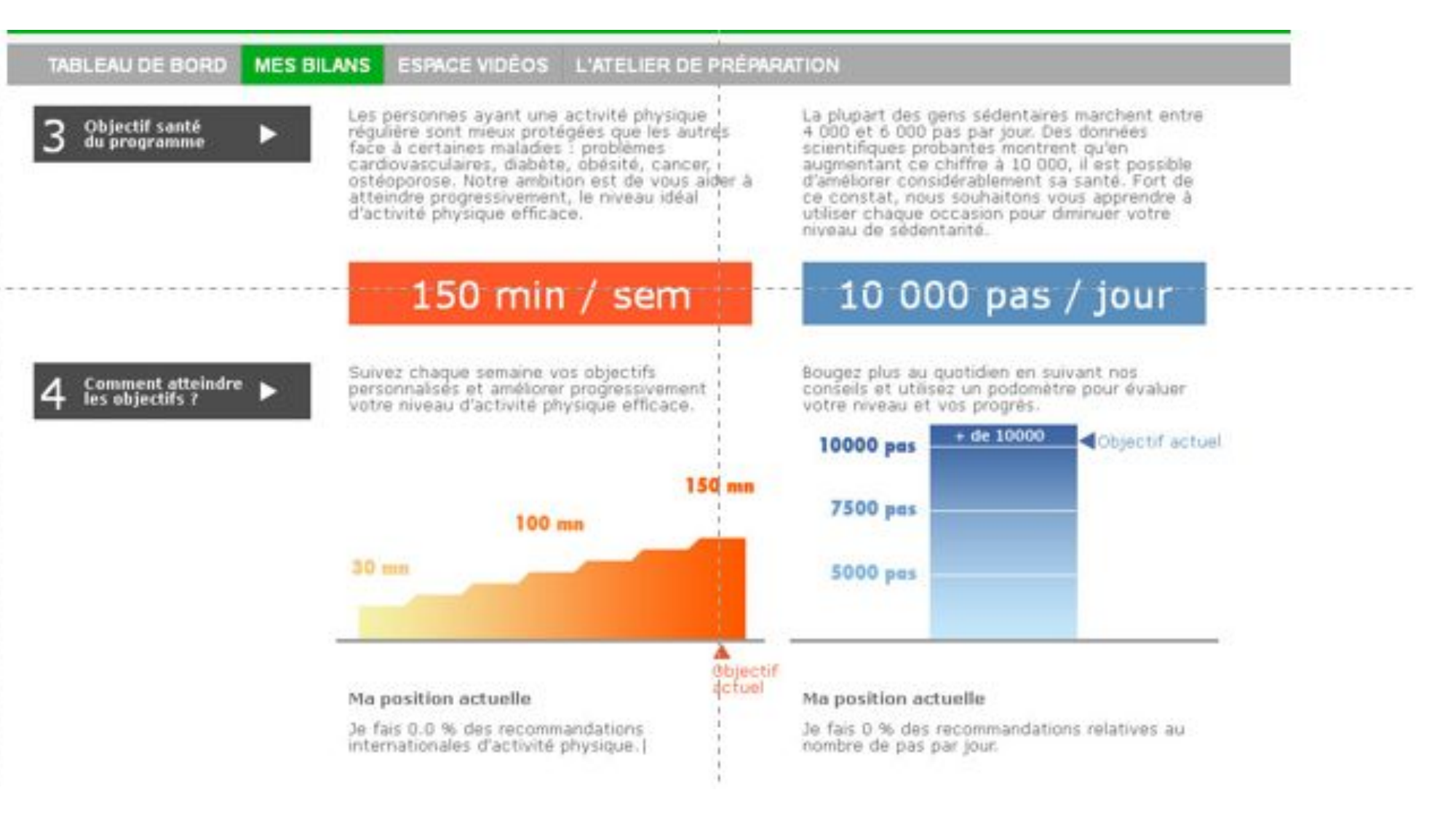

#
G

Supplement: Multimedia Appendix 1 [file jmir_v19i11e360_app1.pptx]
