# Supplementary material for: A Fully Automated Web-Based Program Improves Lifestyle Habits and HbA1c in Patients With Type 2 Diabetes and Abdominal Obesity: Randomized Trial of Patient E-Coaching Nutritional Support (The ANODE Study)
Source: J Med Internet Res. 2017 Nov 8;19(11):e360. doi: 10.2196/jmir.7947 (PMC5700402; doi:10.2196/jmir.7947)
Supplement: Multimedia Appendix 2 [file jmir_v19i11e360_app2.pdf]

Multimedia Appendix 2. Baseline characteristics of the population.

		e-coaching	Control	P-value
Total participants (n)		60	60	
Age, years (SD)		57.6 (8.1)	55.5 (10.3)	.22
Sex, n (%)				1.00
	Male	20 (33.3)	20 (33.3)	
	Female	40 (66.7)	40 (66.7)	
Body weight (kg)		93.3 (16.2)	93.5 (15.0)	.92
BMI, kg/m^2 (SD)		33.4 (4.2)	33.4 (3.3)	.96
Waist circumference, cm (SD)		110 (10)	108 (9)	.46
Systolic blood pressure, mmHg (SD)		133 (15)	133 (12)	.79
VO_2 max, mL/min/kg (SD)		20.8 (6.1)	21.6 (6.0)	.48
HbA1c, % (SD)		7.16 (0.78)	7.27 (0.89)	.47

Fasting blood glucose, mmol/L (SD)		7.58 (1.57)	7.87 (1.76)	.33
Total cholesterol (mg/dl)		183 (42)	177 (43)	.45
LDL-C (mg/dl)		105 (38)	102 (38)	.75
HDL-C (mg/dl)		45 (14)	45 (14)	.93
Triglycerides (mg/dl)		183 (135)	152 (73)	.12
Serum Glutamic Pyruvic Transaminase (IU/l)		29 (14)	33 (14)	.08
Serum Glutamic Oxaloacetic Transaminase (IU/l)		36 (21)	44 (24)	.06
Gammaglutamyl-transferase (IU/l)		46 (30)	48 (25)	.49
hs-CRP (mg/l)		2.79 (1.99)	2.66 (2.09)	.53
Uric acid (µmol/l)		343 (78)	336 (95)	.68
Creatinine (µmol/l)		78 (17)	74 (18)	.23
Dietary intake				

	Energy (kcal/d)	1984 (702)	2132 (688)	.24
	Fibers (g/d)	20 (8)	19 (8)	.29
	Carbohydrates (g/d)	204 (80)	211 (73)	.62
	Proteins (g/d)	85 (29)	96 (31)	.05
	Lipids (g/d)	83 (32)	89 (37)	.33
	Saturated fat (g/d)	32 (14)	36 (17)	.22
	Sodium (mg/d)	3083 (1223)	3503 (1353)	.08
	Calcium (mg/d)	883 (320)	921 (363)	.54
	Empty calories (g/d)	1413 (658)	1609 (668)	.11
	DQI-I (/100)	54.0 (5.7)	52.8 (6.2)	.28
Comorbidity/medications				
Microangiopathy n (%)		1 (1.9)	4 (6.7)	.36

History of cardiovascular disease, n (%)		2 (3.3)	4 (6.7)	.68
Lipid-lowering drugs n (%)		30 (50)	27 (45.8)	.64
Antidiabetic drugs n (%)		55 (91.7)	58 (96.7)	.44
Antihypertensive drugs n (%)		33 (55.0)	30 (50.0)	.58
